# FEM Modeling of the Temperature Influence on the Performance of SAW Sensors Operating at GigaHertz Frequency Range and at High Temperature Up to 500 °C

**DOI:** 10.3390/s20154166

**Published:** 2020-07-27

**Authors:** Jean Claude Asseko Ondo, Eloi Jean Jacques Blampain, Gaston N’Tchayi Mbourou, Stephan Mc Murtry, Sami Hage-Ali, Omar Elmazria

**Affiliations:** 1 Laboratoire Modélisation et Simulation de Composants (LMSC), Faculté des Sciences, USTM, Franceville B.P. 941, Gabon; jeanclaudeasseko@gmail.com (J.C.A.O.); ntchayi@gmail.com (G.N.M.); 2 Institut Jean Lamour, UMR 7198, Université de Lorraine—CNRS, 54000 Nancy, France; stefan.mc-murtry@univ-lorraine.fr (S.M.M.); sami.hage-ali@univ-lorraine.fr (S.H.-A.)

**Keywords:** SAW sensor, FEM modeling, piezoelectric, AlN, sapphire, high temperature

## Abstract

In this work, we present a two-dimensional Finite Element Method (2D-FEM) model implemented on a commercial software, COMSOL Multiphysics, that is used to predict the high temperature behavior of surface acoustic wave sensors based on layered structures. The model was validated by using a comparative study between experimental and simulated results. Here, surface acoustic wave (SAW) sensors consist in one-port synchronous resonators, based on the Pt/AlN/Sapphire structure and operating in the 2.45-GHz Industrial, scientific and medical (ISM) band. Experimental characterizations were carried out using a specific probe station that can perform calibrated measurements from room temperature to 500 °C. In our model, we consider a pre-validated set of physical constants of AlN and Sapphire and we take into account the existence of propagation losses in the studied structure. Our results show a very good agreement between the simulation and experiments in the full range of investigated temperatures, and for all key parameters of the SAW sensor such as insertion losses, resonance frequency, electromechanical factor of the structure (k^2^) and quality factor (Q). Our study shows that k^2^ increases with the temperature, while Q decreases. The resonance frequency variation with temperature shows a good linearity, which is very useful for temperature sensing applications. The measured value of the temperature coefficient of frequency (TCF) is equal to −38.6 ppm/°C, which is consistent with the numerical predictions.

## 1. Introduction

Surface acoustic wave (SAW) devices have been popular for several decades and are used in many fields. They are widely used in the telecommunications industry for the production of filters, delay lines and resonators operating at frequencies ranging from a few tens of megahertz to a few gigahertz [1]. Because of their great sensitivity to external conditions, they have also been the subject of numerous studies relating to their use as micro-sensors for pressure measurement [2], deformations [3], gases and liquids (for the detection of chemical elements) [4,5], magnetic fields [6,7] and temperatures [8]. Among SAW sensors, resonators occupy an important place due to their peculiarity of storing energy when they are electrically excited by a radio frequency (RF) signal. SAW sensors have the advantage of being small and passive. They can be interrogated remotely by recording the backscattered RF response. They have good resistance properties in extreme environments and great sensitivity to the physical parameters to be measured.

For high and very high temperature applications, active sensors are no longer suitable due to the limits of their integrated circuits and the choice is directed towards SAW devices, when wired solutions are not possible. The usual piezoelectric materials (substrates) for SAW devices such as quartz [9] may not be suitable for sensors operating at very high temperatures, and the potential of lithium niobate in congruent [10,11] and stoichiometric [12] formulations requires more investigations. Studies have shown that Langasite (LGS) is able to operate at temperatures above 900 °C without losing its piezoelectric properties [13,14]. However, it has a number of limitations, the most important being the excessive damping (propagation losses) of surface waves when the operating frequency exceeds 1 GHz [15]. Recently, efforts have been devoted to the development of a SAW sensor heterostructure for high and very high temperature applications such as AlN/Pt/LiNbO_3_ [16] and AlN/Sapphire [17,18,19,20,21]. The AlN/Sapphire SAW structure can also operate at very high frequencies [18].

To predict the behavior of a SAW devices in harsh conditions and to design high-performance devices, modeling techniques such as the δ function model [22], the coupling of mode (COM) model [23], the P-matrix model [24,25], and the equivalent circuit model [26,27] have become essential in their design. The Finite Element Method, which can be implemented using commercial software such as COMSOL Multiphysics, has an excellent ability to model and analyze SAW devices and has a simplicity advantage compared to other methods when it comes to multilayer structures, which are the focus of the present study (Pt/AlN/Sapphire).

In this work, we present a two-dimensional Finite Element Method (2D-FEM) model of Pt/AlN/Sapphire SAW one-port resonators for high-temperature and high-frequency sensor applications. The aim is to study the influence of temperature variations on device operation. Elasticity, piezoelectricity, permittivity tensors, the expansion coefficient ($\alpha_{i}$) and density coefficients (ρ) are modified as a function of temperature. The variation between all these parameters is most often given in the form of a Taylor series development [28]. The extraction of the temperature response from several parameters of resonator are analyzed and compared with the experimental results. We will restrict our study to the first Rayleigh mode and the temperature range 23–500 °C.

## 2. Theoretical Model and Simulation Methodology

### 2.1. Theoretical Model

Surface acoustic wave (SAW) devices, in their operation, are governed by differential equations which must be solved by taking into account certain parameters: the geometric complexity of the device, the properties of materials and the boundary conditions. The Finite Element Method (FEM) provides numerical solutions defined by associated differential equations. The equations that make the link between stress, deformation, electric field and electric displacement field in the stress charge of a piezoelectric crystal, are given by [28]:

(1) $$T_{ij}=C_{ijkl}^{E}S_{kl}-e_{ijk}E_{k}$$

(2) $$\;D_{i}=e_{jkl}S_{kl}+\varepsilon_{ij}^{S}E_{j}$$

where, $T_{ij}$ represents the stress vector, $C_{ijkl}$ the elasticity matrix (N/m^2^), $e_{ijk}$ the piezoelectric matrix (C/m^2^), $\varepsilon_{ij}$ the permittivity matrix (F/m), $E_{k}$ the electric field vector (V/m), $S_{kl}$ the strain vector, and $D_{i}$ is the electrical displacement (C/m^2^). 

The piezoelectric resonator is subject to a dissipation of its energy. The resonator is subjected to a set of mechanical, dielectric and piezoelectric losses. In the case of an anisotropic material, we can introduce a viscosity coefficient $\eta_{C}^{m,n}$, a dielectric loss factor $\eta_{\varepsilon }^{m,n}$ and a piezoelectric loss factor $\eta_{e}^{m,n}$ to take into account these different losses. In this case, coefficients $\;C_{ijkl}$, $\varepsilon_{ij}$ et $e_{ijk}$ become complex [29,30,31]:

(3) $${\bar{C}}_{ijkl}^{m,n}=\left(1+j\eta_{C}^{m,n}\right)C_{ijkl}^{m,n}$$

(4) $${\bar{\varepsilon }}_{ij}^{m,n}=\left(1-j\eta_{\varepsilon }^{m,n}\right)\varepsilon_{ij}^{m,n}$$

(5) $${\bar{e}}_{ijk}^{m,n}=\left(1-j\eta_{e}^{m,n}\right)e_{ijk}^{m,n}$$

where *m* and *n* are the components of each tensor.

Variations in temperature in a material produce two main phenomena: the material expands or contracts, and its physical properties change. The elastic ($C_{ijkl}$), piezoelectric ($e_{kij}$), permittivity ($\varepsilon_{kj}$) and density (ρ) coefficients are modified as a function of temperature.

(6) $$C_{ijkl}\left(T\right)=C_{ijkl}\left(T_{0}\right)\left[1+\theta_{1}^{C}\left(T-T_{0}\right)+\theta_{2}^{C}{\left(T-T_{0}\right)}^{2}+\cdots \right]$$

(7) $$e_{kij}\left(T\right)=e_{kij}\left(T_{0}\right)\left[1+\theta_{1}^{e}\left(T-T_{0}\right)+\theta_{2}^{e}{\left(T-T_{0}\right)}^{2}+\cdots \right]$$

(8) $$\varepsilon_{kj}\left(T\right)=\varepsilon_{kj}\left(T_{0}\right)\left[1+\theta_{1}^{\varepsilon }\left(T-T_{0}\right)+\theta_{2}^{\varepsilon }{\left(T-T_{0}\right)}^{2}+\cdots \right]$$

(9) $$\rho \left(T\right)=\rho \left(T_{0}\right)\left[1+\theta_{1}^{\rho }\left(T-T_{0}\right)+\theta_{2}^{\rho }{\left(T-T_{0}\right)}^{2}+\cdots \right]$$

Thermal expansion coefficients ($\alpha_{i}$) are also sensitive to temperature. They describe the expansion dl/l in three spatial directions. Variations in these parameters are most often given in the form of a Taylor series expansion [28]:

(10) $$\alpha_{i}\left(T\right)=\alpha_{i}\left(T_{0}\right)\left[1+\theta_{1}^{\alpha_{i}}\left(T-T_{0}\right)+\theta_{2}^{\varepsilon }{\left(T-T_{0}\right)}^{2}+\cdots \right]$$

where:

$$\theta_{1}^{C}=\frac{1}{C_{ijkl}}\frac{dC_{ijkl}}{dT},\;\theta_{2}^{C}=\frac{1}{2}\frac{1}{C_{ijkl}}\frac{d^{2}C_{ijkl}}{dT^{2}},\;\theta_{1}^{e}=\frac{1}{e_{kij}}\frac{de_{kij}}{dT},\;\theta_{2}^{e}=\frac{1}{2}\frac{1}{e_{kij}}\frac{d^{2}e_{kij}}{dT^{2}},$$

$$\theta_{1}^{\varepsilon }=\frac{1}{\varepsilon_{kj}}\frac{d\varepsilon_{kj}}{dT},\;\theta_{2}^{\varepsilon }=\frac{1}{2}\frac{1}{\varepsilon_{kj}}\frac{d^{2}\varepsilon_{kj}}{dT^{2}},\;\theta_{1}^{\rho }=\frac{1}{\rho }\frac{d\rho }{dT},\;\theta_{2}^{\rho }=\frac{1}{2}\frac{1}{\rho }\frac{d^{2}\rho }{dT^{2}},\;\theta_{1}^{\alpha_{i}}=\frac{1}{\alpha_{i}}\frac{d\alpha_{i}}{dT},\;\theta_{2}^{\alpha_{i}}=\frac{1}{2}\frac{1}{\alpha_{i}}\frac{d^{2}\alpha_{i}}{dT^{2}},$$

The coefficients ($\theta_{1}^{C},\theta_{2}^{C}$), ($\theta_{1}^{e},\theta_{2}^{e}$), ($\theta_{1}^{\varepsilon },\theta_{2}^{\varepsilon }$), ($\theta_{1}^{\rho },\theta_{2}^{\rho }$) and ($\theta_{1}^{\alpha_{i}},\theta_{2}^{\alpha_{i}}$) are, respectively, the first and second order temperature constants of stiffness, piezoelectricity, permittivity, density and thermal expansion. They are determined experimentally, but obtaining them is difficult and time-consuming work. In practice, it is considered that the dielectric and piezoelectric constants do not vary with temperature [28,32].

### 2.2. Simulation Methodology

**Figure 1:**
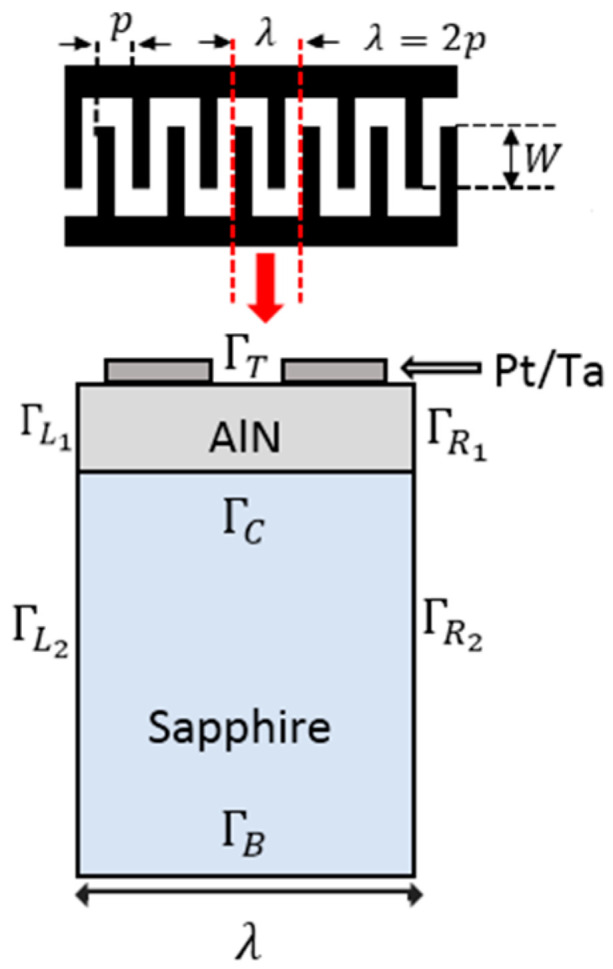
Two-dimensional one-period representation of Pt/AlN/Sapphire structure.

COMSOL Multiphysics allows us to realize the geometry of the two-dimensional model (2D) corresponding to one period of the periodic infinite structure SAW Pt-Ta/AlN/Sapphire (Figure 1). The plane deformation conditions must be specified for each element, by imposing that all the gradients in the third direction must be supposed to be null. The Platinum electrodes and the tantalum adhesion layer are deposited on the piezoelectric layer of aluminum nitride with c-axis orientation and the non-piezoelectric substrate is made of Sapphire and also oriented to the “c-axis”. The dimensions of the studied structure are presented in Table 1.

The material properties, which have to be considered in a piezoelectric analysis of SAW resonators, belong to three categories: mechanical, electrical and piezoelectric. Mechanical properties have to be defined for all three materials, Platinum (electrodes), AlN (piezo layer) and Sapphire (substrate), but electrical and piezoelectric properties have to be defined only for the AlN layer. The properties of the substrate, the piezoelectric layer, and the Platinum electrodes are inserted as matrices in COMSOL. In the same way, material properties such as density (ρ=16,600 kg/m^3^), Young’s modulus (E=175 GPa) and the Poisson’s coefficient (σ=0.34) should be specified for Tantalum. All physical constants used in this work are shown in Table 2. Table 3 presents the first order temperature coefficients of different physical constants used in our simulation for taking into account the temperature effect on our resonator.

The values of the mechanical, dielectric and piezoelectric loss factors used in our simulations are given by Equations (11)–(13).

(11) $$\eta_{C}^{m,n}=\left|\begin{array}{ccc} 4 & 3 & 3.5\\ 3 & 4 & 3.5\\ \begin{array}{c} 3.5\\ 0\\ \begin{array}{c} 0\\ 0 \end{array} \end{array} & \begin{array}{c} 3.5\\ 0\\ \begin{array}{c} 0\\ 0 \end{array} \end{array} & \begin{array}{c} 4.5\\ 0\\ \begin{array}{c} 0\\ 0 \end{array} \end{array} \end{array}\;\;\;\begin{array}{ccc} 0 & 0 & 0\\ 0 & 0 & 0\\ \begin{array}{c} 0\\ 2.3\\ \begin{array}{c} 0\\ 0 \end{array} \end{array} & \begin{array}{c} 0\\ 0\\ \begin{array}{c} 2.3\\ 0 \end{array} \end{array} & \begin{array}{c} 0\\ 0\\ \begin{array}{c} 0\\ 1.7 \end{array} \end{array} \end{array}\right|\times 10^{-3}$$

(12) $$\eta_{\varepsilon }^{m,n}=\left|\begin{array}{ccc} 8 & 0 & 0\\ 0 & 8 & 0\\ 0 & 0 & 6.7 \end{array}\right|\times 10^{-3}$$

(13) $$\eta_{e}^{m,n}=\left|\begin{array}{ccc} 0 & 0 & 0\\ 0 & 0 & 0\\ 9.2 & 9.2 & 1 \end{array}\;\;\;\;\begin{array}{ccc} 0 & 9.9 & 0\\ 9.9 & 0 & 0\\ 0 & 0 & 0 \end{array}\right|\times 10^{-3}$$

The application of boundary conditions is the most critical part of the finite element resolution. Within the seven limits ($\Gamma_{T}$,${\;\Gamma }_{L1}$, $\Gamma_{L2}$, $\Gamma_{R1}$, $\Gamma_{R2}$, $\Gamma_{C}$, $\Gamma_{B}$), $\Gamma_{T}$ is the free boundary condition,${\;\Gamma }_{B}$ is the Dirichlet boundary condition, $\Gamma_{R}$ is the right periodic boundary condition, $\Gamma_{L}$ is the left periodic boundary condition and $\Gamma_{C}$ is the condition of the Sapphire–AlN interface.

Free boundary conditions $\Gamma_{T}$ must be specified on the top of the substrate and electrodes—that is, there should be no mechanical stress on this surface. The Dirichlet boundary condition is applied on the lower surface $\Gamma_{B}$, with displacements which cancel out on this border. The periodic boundary conditions are applied at the levels of the left and right limits according to [38]:

(14) $$\Gamma_{R}\left(u_{i},V\right)=\eta \Gamma_{L}\left(u_{i},V\right)$$

where $\eta ={\left(-1\right)}^{n}$, $u_{i}$ is the displacement, *V* is the potential, n=2L/λ, L is the width of along the direction of the wave propagation. In the simulation, the value of *n* is 2. Table 4 presents the boundary condition details.

COMSOL Multiphysics ensures an automatic mesh which allows the user to adapt the shape and dimensions of meshes to the geometric model of a structure. It also allows the user to make a customized mesh. A preliminary mesh optimization study is therefore necessary to find a compromise between calculation time and precision [39]. In our simulation we chose a manual mesh. All modeling was done on a HP ProDesk 400 G3 core i5 (processor: 3.2 GHz; memory: 8 GB RAM) computer.

## 3. Experimental Details

To assess the accuracy of our simulation using the FEM, we fabricated a one-port SAW resonator on Pt/AlN/Sapphire with the same geometry. The fabrication process and the characterization method are described below.

Our single-port resonator consists of a c-axis oriented Sapphire substrate on which a 1-μm-thick epitaxial layer of AlN is deposited by the Metal Organic Chemical Vapor Deposition (MOCVD) technique. This thin layer of AlN has a very low roughness (RMS = 7.8 Å) and is highly textured. Above this AlN film, 10 nm of tantalum thickness is deposited (adhesion layer) and 90 nm of Platinum, by the RF magnetron sputtering technique, for the manufacture of interdigital transducers (IDT). The minimum line width was ~0.4 μm to meet a center frequency of 2.45 GHz. The IDT and reflectors were fabricated using electron beam lithography and the ion beam etching technique (IBE). The acoustic wavelength of the device is defined by the spatial period of the IDT and was fixed to 1.7 μm. The thickness of the AlN film (h_AlN_) was chosen to have a sufficiently large electromechanical coupling (k^2^). Indeed, the dispersion curve of k^2^ with the h_AlN_/λ ratio shows that the value of k^2^ increases to reach a plateau from h_AlN_/λ = 0.4 [32]. In our case, h_AlN_/λ is set to 0.58 to ensure a safety margin due to the specific properties of Platinum.

The frequency responses of SAW devices and the reflection ($S_{11}$) mode, were measured using a Network Analyzer (Agilent PNA 5230A, Santa Clara, CA, USA) and RF prober station (Signatone S-1160, Gilroy, CA, USA) equipped with a Signatone Thermal probing system S-1060 Series, to withstand temperatures up to 600 °C. The RF probe was equipped with a water-cooling system to withstand higher temperatures than a conventional one. Our experiments were done in the range from 23 °C to 500 °C.

The measurement of reflection coefficient $S_{11}$ makes it possible to know the central frequency of the structure; this corresponds to the minimum value of $S_{11}$. The measurement of $S_{11}$ also makes it possible to extract the functional parameters of the SAW devices such as the phase velocity *v*, the electromechanical coupling coefficient k^2^, and the quality factor Q.

There is a relationship between the reflection coefficient $S_{11}$ and the electrical impedance Z [1] given by Equation (15):

(15) $$Z=Z_{0}\frac{1+S_{11}}{1-S_{11}}$$

where $Z_{0}$ is the reference impedance. In practice, it is usually 50 Ω.

## 4. Results and Discussion

### 4.1. Reflection Coefficient S_11_ and Impedance Z, Behavior

**Figure 2:**
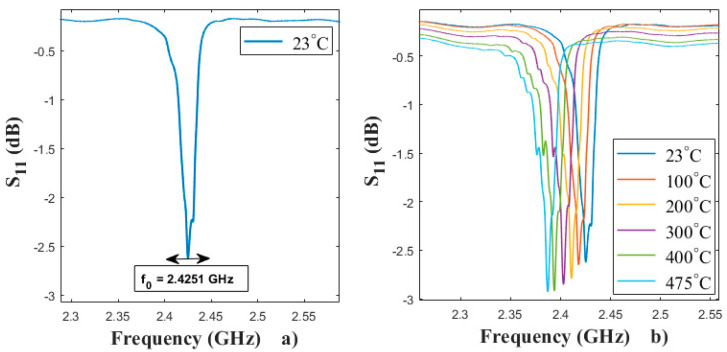
(**a**): Measured reflection coefficient $S_{11}$ of the Pt/AlN/Sapphire sensor at 23 °C, (**b**): evolution of the reflection coefficient as a function of the temperature.

The narrow-band frequency response of $S_{11}$, ranging from 2.25 GHz to 2.6 GHz, was measured as shown in Figure 2a The center frequency of the first mode measured at room temperature (RT) and is equal to 2.4251 GHz. The frequency responses of SAW devices, reflection ($S_{11}$) mode, were measured in the temperature range 23–500 °C and are shown in Figure 2b We note a slight decrease in the Pt/AlN/Sapphire resonator amplitude peak and a decrease in frequency with increasing temperature.

In the modeled structure, we used an Eigen frequency analysis to determine the Eigen frequencies and the modes of deformation. A frequency domain study was used to calculate the response of our model when subjected to harmonic excitation for one or more frequencies. Through the use of simulations, we were able to determine the wave types by observing the vibration of the wave in the structure. The model was meshed manually. The SAW displacements are the largest near the AlN layer surface, and the meshing domain is meshed to higher densities near the surface rather than near the bottom.

**Figure 3:**
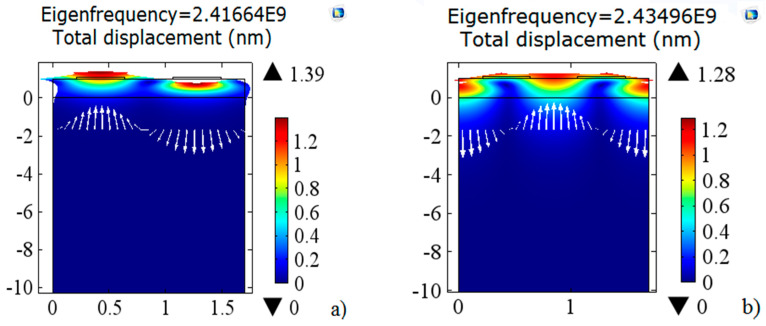
Rayleigh wave mapping of the first two symmetrical (**a**) and antisymmetric (**b**) modes of 23 °C simulated Pt/AlN/Sapphire structure.

Figure 3 shows the mappings of the first two modes of symmetrical (Figure 3a) and antisymmetric (Figure 3b) Rayleigh waves in the Pt/AlN/Sapphire structure, simulated at 23 °C. The Eigen frequency of the symmetrical mode noted that $f_{sc+}$ is equal to 2.41664 GHz and the corresponding amplitude of displacement is 1.36449 nm. The Eigen frequency of the asymmetric mode noted that $f_{sc-}$ is equal to 2.43496 GHz and the corresponding amplitude of displacement is 1.27282 nm.

The electrical impedance *Z* is frequently used in SAW analysis. Its modulus makes it possible to highlight the resonance and anti-resonance phenomena associated with the frequencies $f_{r}$ (minimum value of *Z* module) and $f_{a}$ (maximum value of *Z* module). It can be defined by [39,40]:

(16) $$Z=\frac{V}{jwq}$$

where *V* is the electric potential, *w* the angular frequency and *q* the electric charge.

**Figure 4:**
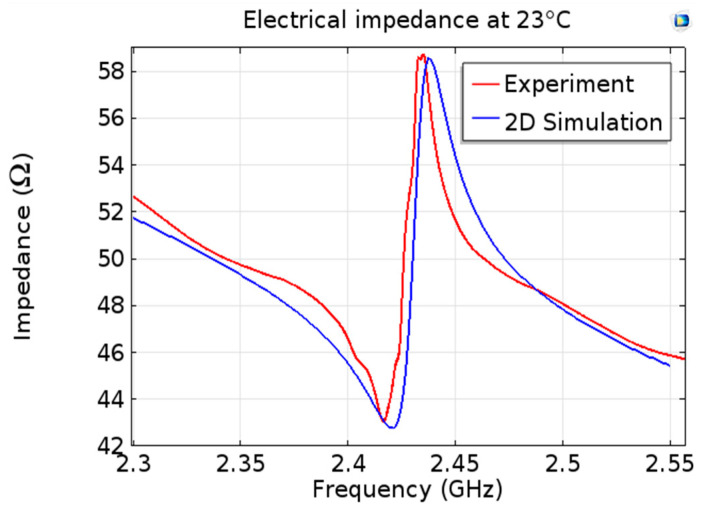
Comparison of the simulated and experimental impedance moduli at 23 °C.

We can access in COMSOL the modeled values of the electric potential as a function of the frequency and consequently obtain modeled module of impedance as a function of temperature. Figure 4 shows simulated (blue) and experimental (red) impedance as a function of the frequency at 23 °C. We can see a good agreement between the two curves. The simulated values of frequencies $f_{r}$ and $f_{a}$ are, respectively, 2.4166 GHz and 2.4349 GHZ.

**Figure 5:**
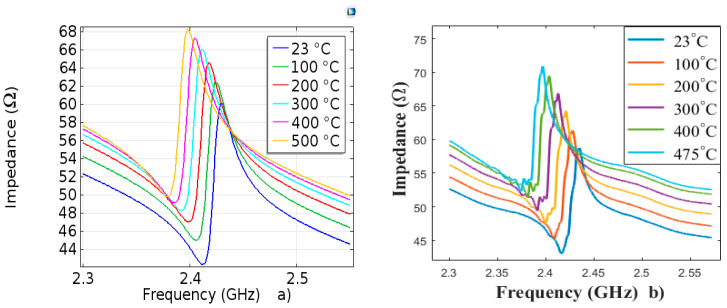
Variations in impedance as a function of temperature: (**a**) simulation (**b**) measurement.

The impedance variations as a function of temperature for simulated and measured devices are shown in Figure 5, in the temperature range [23 °C, 500 °C].

We observe that the two responses of impedance with temperature are similar.

### 4.2. Rayleigh Phase Velocity

The phase velocity is defined by:

(17) $$V_{SAW}=f\times \lambda$$

where λ is the acoustic wavelength equal to the spatial period of the IDTs and $f=\left(f_{sc+}+f_{sc-}\right)/2$.

**Figure 6:**
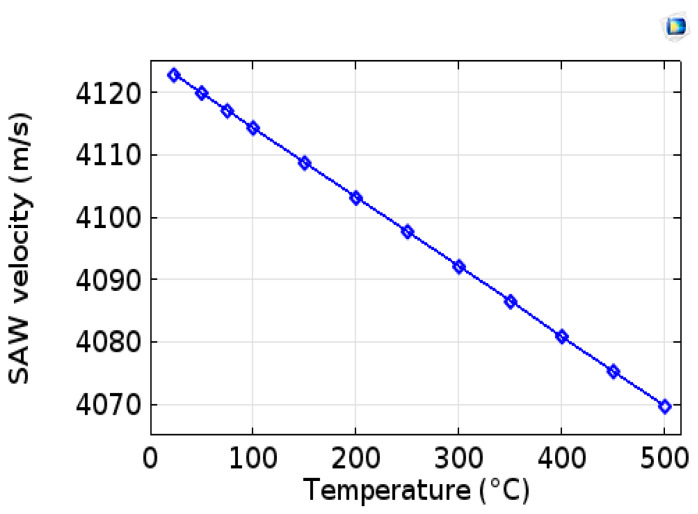
Variation in Rayleigh velocity as a function of temperature.

The phase velocity resulting from the simulation is 4123.86 m/s and that obtained experimentally is 4123.52 m/s. These two velocities are in very close agreement. On the other hand, these velocities are very low compared to 5600 m/s expected for the free surface of the AlN/Sapphire structure. This is mainly due to mass loading of Platinum IDT. The Rayleigh velocity curve as a function of the temperature obtained by simulation is shown in Figure 6. We observe a drift in the phase velocity with the increase in temperature. This behavior is similar to the experimental literature results.

### 4.3. Electromechanical Coefficient Factor k^2^

The electromechanical coupling factors k^2^ indicate the effectiveness with which a piezoelectric material converts electrical energy into mechanical energy or vice versa. The k^2^ was determined using measured and simulated data for the $S_{11}$ parameter (reflection) and the following Equation (18) derived from [18]:

(18) $$k^{2}\left(\%\right)=\frac{\pi }{2}\left(\frac{f_{r}}{f_{a}}\right)tan\left[\frac{\pi }{2}\left(\frac{f_{a}-f_{r}}{f_{a}}\right)\right]\times 100$$

**Figure 7:**
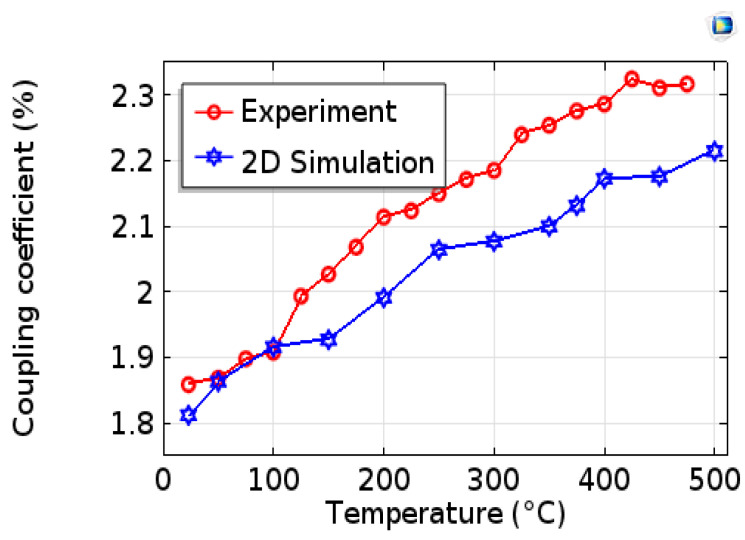
Evolution of experimental and simulated electromechanical coefficients versus temperature.

At room temperature (23 °C), the value of the electromechanical coupling coefficient of our modeled device is 1.812% and the measured value is 1.868%. Figure 7 presents a comparison of the variation in electromechanical coupling as a function of the temperature obtained from the experimental measurements and the simulations of the Pt/AlN/Sapphire sensor [18]. Figure 7 shows that the k^2^ coefficient increases with temperature. For measured and modeled k^2^, enhancements of 27% and 22%, respectively, are recorded when the temperature increase from 23 to 500 °C. This demonstrates that the piezoelectrical properties of the AlN/Sapphire structure are enhanced by temperatures up to at least 500 °C. Note that when the device is cooled down, k^2^ returns to its original value. We observe a good agreement of the two dispersion curves of k^2^. We note, however, that k^2^ resulting from the simulation increases more slowly with temperature than that obtained experimentally.

### 4.4. Quality Factor Q

**Figure 8:**
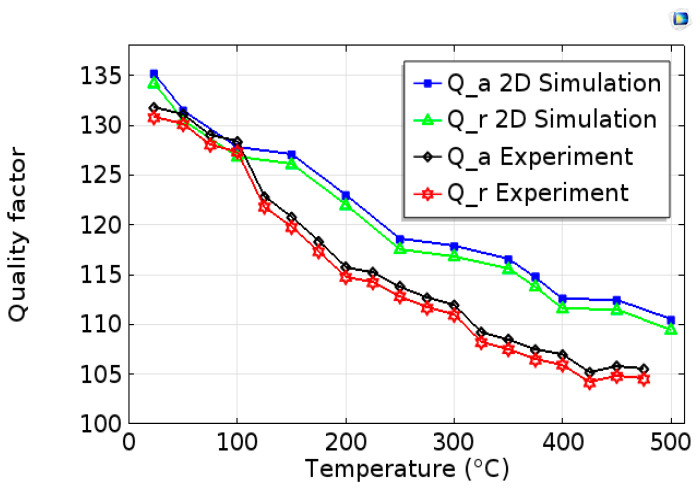
Evolution of the quality factor Q as a function of temperature for experimental and simulated devices, for resonance and anti-resonance frequencies.

The quality factor (Q) extracted from the impedance frequency curves using (17) is plotted in Figure 8 versus temperature. We can define the resonance ($Q_{r}$) and anti-resonance ($Q_{a}$) quality factors by [18]:

(19) $$Q_{i}=\left|\frac{f_{i}}{f_{a}-f_{r}}\right|$$

where i≡{r,a}.

At room temperature, we obtain the following values: for measurements $Q_{r}=130.76$ and $Q_{a}=131.76$, for simulations $Q_{r}=134.17$ and $Q_{a}=135.17$. One can also observe that the quality factors modelled and measured decrease (by about 23%) when temperature increases. Note that the quality factor is inversely proportional to the ratio of the dissipated energy to the stored energy, which is represented by the loss factor (tan δ) in dynamic mechanical analysis (DMA).

### 4.5. Temperature Coefficient of Frequency TCF

**Figure 9:**
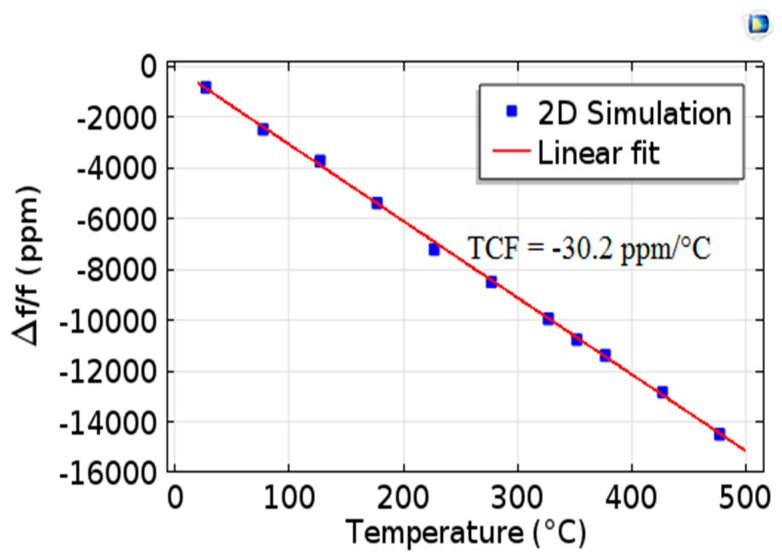
Relative evolution of the resonant frequency as a function of temperature.

To evaluate the potential of the structure as a temperature sensor, the frequency-versus-temperature behavior of the Pt/AlN/Sapphire SAW resonator was investigated. For the 2D simulation, the relative variation in the resonant frequency as a function of temperature is presented in Figure 9. We determined the TCF from the following Equation (20):

(20) $$TCF\left(\mathrm{ppm}/{}^{\circ}C\right)=\frac{1}{\Delta T}\frac{f_{r_{i}}-f_{r}}{f_{r}}\times 10^{6}$$

where $f_{r}$ and $f_{r_{i}}$ are the resonance frequencies measured at 23 °C and at 23 °C + ΔT, respectively. The simulated SAW resonator is characterized by a high and almost constant temperature sensitivity (good linearity at first approximation), with a TCF value of −30.2 ppm/°C. The experimental TCF is approximately equal to −38.6 ppm/°C.

### 4.6. Mechanical Displacements

**Figure 10:**
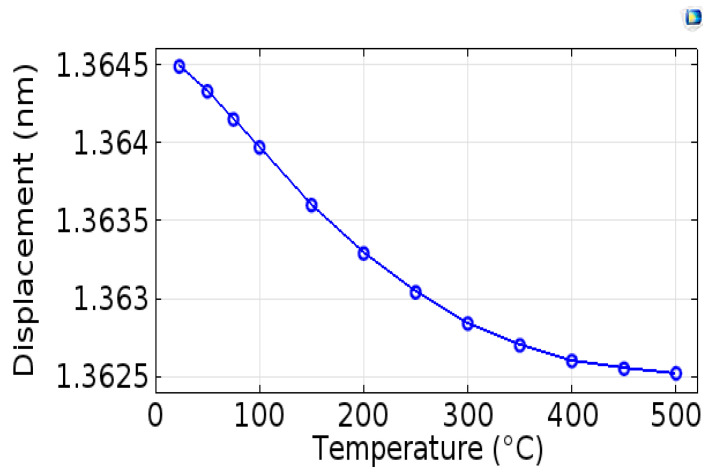
Evolution of mechanical displacements as a function of the temperature obtained by simulation.

After the determination of electromechanical coupling factor k^2^, quality factor Q, and the temperature coefficient of frequency TCF of the structure, we studied, by simulation, variations in the total mechanical displacements in the AlN layer as a function of temperature. The result is presented in Figure 10. We note that the amplitude of mechanical displacements in the AlN layer decrease with increasing temperature. The decrease in the displacements’ amplitude can be justified by the decrease in the signal observed experimentally.

## 5. Conclusions

In this work, we modeled, by the Finite Element Method in 2D, the influence of temperature on the performance of a Pt/AlN/Sapphire SAW one-port resonator, which was used to predict the high-temperature behavior of surface acoustic wave sensors. In our model, we considered a pre-validated set of physical constants of AlN and Sapphire and we took into account the existence of propagation losses in the studied structure. Experimental characterizations were carried out using a specific probe station that can perform calibrated measurements from room temperature to 500 °C. The comparison of the modeled and measured curves of $S_{11}$ and impedance *Z* as a function of temperature showed very good agreement, thus validating our simulation methodology. The influence of the temperature on the functional parameters of the device such as the phase velocity of the Rayleigh wave, the electromechanical coupling coefficient k^2^, the quality factor Q and the temperature coefficient of frequency (TCF) was modeled and compared with the experimental results. Overall, there is very good agreement between the simulated and experimental results in our temperature study range (23–500 °C). The phase velocity resulting from the simulation, 4123.86 m/s, and that obtained experimentally, 4123.52 m/s, are very close. The k^2^ coupling coefficients increase with temperature; for measured and modeled k^2^ coupling coefficients, enhancements of 27% and 22%, respectively, were recorded in our temperature study range. It can also be noted that the quality factors modeled and measured decrease by around 23% when the temperature increases in this temperature range. The relative variation in the resonant frequency TCF as a function of temperature is characterized by a good linearity at first approximation, which is very useful for temperature sensing applications, with TCF values of −30.2 ppm/°C and −38.6 ppm/°C, respectively, for modeled and measured devices. Finally, this work shows us that a thorough investigation of the influence of temperature on electronic devices can be performed by 2D Finite Element Method modeling, with very good precision.

In our next work, we will show how to improve the sensitivity (TCF) of this bilayer resonator, using an appropriate geometry modification of its structure. In fact, Platinum IDTs can be buried in the AlN piezoelectric layer to achieve this.

## Figures and Tables

**Table 1:** Sizing of Pt/AlN/Sapphire structure.

Parameters	Settings
Wavelength (2p)	1.7 µm
Platinum electrode thickness	90 nm
Tantalum adhesion layer	10 nm
Metallization ratio	1/2
Aperture (W)	30 µm
Thickness of AlN c-axis layer	1 µm
Thickness of substrate c-axis	50 µm

**Table 2:** Physical constants of AlN, Sapphire and Platinum.

Material	Symbol	AlN [33]	Sapphire [34]	Platinum [35]
Density (kg/m^3^)	ρ	3260	3980	21,450
Elastic constants (GPa)	$C_{11}$	345	497	348.0
	$C_{12}$	125	164	241.74
	$C_{13}$	120	111	-
	$C_{14}$	-	−23.5	-
	$C_{33}$	395	498	-
	$C_{44}$	118	147	-
Piezoelectric constants (C/m^2^)	$e_{15}$	−0.48	-	-
	$e_{31}$	−0.58	-	-
	$e_{33}$	1.55	-	-
Dielectric constants (10^−11^ F/m)	$\varepsilon_{11}$	8.2	2.25	-
	$\varepsilon_{33}$	9.5	10.2	-

**Table 3:** First order temperature coefficient values of physical constants of AlN and Sapphire.

Material	Symbol	AlN [36]	Sapphire [37]
Density temperature coefficient (10^−6^/°C)	$T_{\rho }$	−14.69	-
Temperature coefficient of elastic constants (10^−4^/°C)	$T_{C_{11}}$	0.8	−0.75
	$T_{C_{12}}$	1.8	0.4
	$T_{C_{13}}$	1.6	−0.8
	$T_{C_{14}}$	-	−0.7
	$T_{C_{33}}$	1	−0.85
	$T_{C_{44}}$	0.5	−1.8
Temperature coefficient of piezoelectric constants (10^−4^/°C)	$T_{e_{15}}$	-	0
	$T_{e_{31}}$	-	0
	$T_{e_{33}}$	-	0
Temperature coefficient of dielectric constants (10^−4^/°C)	$T_{\varepsilon_{11}}$	-	-
	$T_{\varepsilon_{33}}$	-	-
Thermal expansion Coefficients (ppm/°C)	$\alpha_{11}$	5.2	7.2
	$\alpha_{33}$	4.15	8.11

**Table 4:** Boundary conditions.

Boundary	Mechanical Boundary Conditions		Electrical Boundary Conditions
$\Gamma_{T}$	Free		Zero electrical charge
$\Gamma_{C}$	Free		Continuity
$\Gamma_{B}$	Fixed Ground		
$\Gamma_{R1}\;\;\;\;\Gamma_{R2}$ $\Gamma_{L1}\;\;\;\;\Gamma_{L2}$		Periodical boundary conditions	
